# The reciprocal relationship between executive function and theory of mind in middle childhood: a 1-year longitudinal perspective

**DOI:** 10.3389/fpsyg.2014.00655

**Published:** 2014-06-24

**Authors:** Gina Austin, Karoline Groppe, Birgit Elsner

**Affiliations:** Developmental Psychology, Department of Psychology, University of PotsdamPotsdam, Germany

**Keywords:** executive function, theory of mind, longitudinal, middle childhood, attention shifting, inhibition, working memory updating

## Abstract

There is robust evidence showing a link between executive function (EF) and theory of mind (ToM) in 3- to 5-year-olds. However, it is unclear whether this relationship extends to middle childhood. In addition, there has been much discussion about the nature of this relationship. Whereas some authors claim that ToM is needed for EF, others argue that ToM requires EF. To date, however, studies examining the longitudinal relationship between distinct subcomponents of EF [i.e., attention shifting, working memory (WM) updating, inhibition] and ToM in middle childhood are rare. The present study examined (1) the relationship between three EF subcomponents (attention shifting, WM updating, inhibition) and ToM in middle childhood, and (2) the longitudinal reciprocal relationships between the EF subcomponents and ToM across a 1-year period. EF and ToM measures were assessed experimentally in a sample of 1,657 children (aged 6–11 years) at time point one (t1) and 1 year later at time point two (t2). Results showed that the concurrent relationships between all three EF subcomponents and ToM pertained in middle childhood at t1 and t2, respectively, even when age, gender, and fluid intelligence were partialled out. Moreover, cross-lagged structural equation modeling (again, controlling for age, gender, and fluid intelligence, as well as for the earlier levels of the target variables), revealed partial support for the view that early ToM predicts later EF, but stronger evidence for the assumption that early EF predicts later ToM. The latter was found for attention shifting and WM updating, but not for inhibition. This reveals the importance of studying the exact interplay of ToM and EF across childhood development, especially with regard to different EF subcomponents. Most likely, understanding others’ mental states at different levels of perspective-taking requires specific EF subcomponents, suggesting developmental change in the relations between EF and ToM across childhood.

## INTRODUCTION

A major achievement of early development occurs when a child is able to impute mental states to himself/herself and others in order to predict and explain behavior (“theory of mind,” ToM; Frith and Frith, 1999). This ability enables the individual to function in social groups and thus constitutes a crucial aspect of social competence. The development of ToM starts when the child is an infant and continues right the way through to the adolescent years (Lalonde and Chandler, 2002).

A critical test for ToM is the *first-order* *false-belief* *task* which is mastered at around the age of 4 years. One classical task requires the child to infer which belief a character in a story has about the location of an object which has been hidden in the character’s presence, and has then been hidden somewhere else without the character knowing this (Wimmer and Perner, 1983). As children progress through childhood, they are able to solve more complex, so-called higher-order ToM tasks. One of the most commonly used is the *second-order false-belief task* (Perner and Wimmer, 1985) which requires the child to infer a story character’s belief about another person’s belief. The age of mastering second-order false-belief ranges from about 6–7 years, depending on the sample and method used (for a review see Miller, 2009).

Several related abilities of ToM have been identified of which executive function (EF) in particular has received considerable investigation and has led to much theoretical discussion. EF refers to an array of different processes relating to self-control. They develop in the preschool years and continue to do so right up to adolescence (Zelazo and Carlson, 2012). These processes enable the control of thought, action, and emotion, and they include overlapping but distinct EF subcomponents such as attention shifting, inhibition, and updating of working memory (WM updating; Miyake et al., 2000). As regards these specific EF subcomponents, ToM (first-order false-belief) understanding appears to require the ability to suppress one’s own knowledge about reality (inhibition) in order to be able to put oneself into the shoes of the other (attention shifting) and then actively hold the key elements of the story in mind where this information can be monitored and updated in order to make an inference (WM updating; Doherty, 2009).

There is robust evidence that links ToM, (especially first-order false-belief) and these aforementioned specific EF subcomponents – including inhibition (Hughes, 1998a; Carlson and Moses, 2001; Flynn et al., 2004), attention shifting (Frye et al., 1995; Hughes, 1998a), and WM updating (Davis and Pratt, 1995; Keenan et al., 1998) in children aged 3–5 years (see Perner and Lang, 1999, for a review). Several reasons for this relationship have been put forward. For example, EF and ToM make major developments during the preschool years, they seem to share a common neurological basis (prefrontal cortex), and individuals suffering from autism show deficiencies in both (Carlson and Moses, 2001; Hill, 2004).

In need of clarification is whether the robust relationship between EF and ToM found in preschoolers extends to older children. Although there is some evidence that more advanced EF and ToM measures do show positive associations, more studies are needed to confirm this (Miller, 2009). For instance, it has been found that the EF–ToM relationship extends to children between 4^1^/_2_ and 6^1^/_2_ years for second-order false-belief tasks and more demanding EF tasks (Perner et al., 2002). Similar results have been reported for children of middle childhood (Yang et al., 2009; Calderon et al., 2010) and in adolescents (Vetter et al., 2013). However, results in a sample of 8^1^/_2_ year-olds with and without attention deficit hyperactivity disorder (ADHD) were less conclusive (Charman et al., 2001). A correlation between EF and ToM was found in the control group (typically developing children) but as soon as age and intelligence were partialled out, the two constructs were no longer significantly correlated. These inconsistent results show that more studies are needed to clarify the relationship between EF and ToM in older children. This is of particular importance in order to understand whether both constructs remain intertwined in the course of development. It may well be that the link between ToM and EF is less relevant once sufficient cognitive capacities have developed, thereby making it less relevant to regulate one’s own knowledge and view of the world when inferring others’ mental states. Therefore, the first aim of the current study was to investigate whether the EF–ToM relationship extends to middle childhood, and especially, how different EF subcomponents (i.e., attention shifting, WM updating, inhibition) are related to ToM at this age.

Furthermore, an important and controversial question that remains unresolved concerns the causal direction of effect between EF and ToM. Perner (1998; Perner and Lang, 1999, 2000; see also Carruthers, 1996) and Russell (1996, 1997; see also Pacherie, 1997) both maintain that a functional dependency between the two constructs exists but they make opposite predictions as regards the direction of effect.

Perner (1998) claim that the ability to represent mental states on a meta-level is needed for the development of executive control, i.e., ToM enhances EF. In other words, this meta-representational account claims that children need to have a sufficiently developed understanding of their own minds before they will be able to engage in executive control. Russell’s (1996, 1997) theory, on the other hand, claims the exact opposite, i.e., EF is a prerequisite for the emergence of ToM understanding. According to this view, EF is necessary in order to distance oneself from reality and move toward abstract mental states (ToM).

An explanation as to why there is little agreement on the causal relationship between EF and ToM lies in the fact that many studies have based their conclusions on correlational data. There are three types of evidence in order to progress beyond correlational studies (Miller, 2009). First, by means of longitudinal studies (if A predicts B, A must start developing before B). Second, by means of dissociation studies (if A causes B, then A should occur without B; but the opposite should not take place). Third, by means of training studies (if A is trained, what effect, if any, does it have on B?).

There is empirical support for all three types of evidence. First, although there are still relatively few studies examining the longitudinal relationship between EF and ToM performance, there has been a recent increase in the amount of research carried out (Hughes, 2011), including different ages and time spans ranging from very short intervals in so-called microgenetic studies, a method in which the process of developmental change is closely observed and analyzed trial-by-trial, (e.g., Flynn et al., 2004; Flynn, 2007) to longer intervals of up to 1 year (Carlson et al., 2004; Hughes and Ensor, 2007). These studies have mostly been conducted in the preschool or late toddlerhood years. A general finding at this early age is that stronger support is found for the proposal that early EF predicts later ToM than for the view that early ToM predicts later EF. For instance, one of the earliest studies showed that early EF performance (in particular inhibitory control) at age 4 predicted later ToM performance (1 year later), but the reverse was not true (Hughes, 1998b). In a study conducted with 2-year-olds, this pattern of early EF predicting later ToM (15 months later) persisted even when age, gender, and verbal ability were taken into account (Carlson et al., 2004). However, different ToM tasks were used at the two points of measurement, and therefore it is questionable whether the same construct was measured at each time point. This assumption is supported by the finding that the two ToM measures did not correlate over time. Yet, a longitudinal study that included three time points (time intervals ranging from 9 to 12.5 months), also found evidence for the view that earlier EF predicted later ToM in 2-, 3-, and 4-year-olds (Hughes and Ensor, 2007). Others have found similar results (Jahromi and Stifter, 2008; Müller et al., 2012).

However, other longitudinal studies do not support the view that early EF predicts later ToM. For instance, Schneider et al. (2005) in a study including three time points did not find predictive relationships between EF and ToM in either direction after controlling for age and language (Schneider et al., 2005). As noted by the authors, one reason for this finding might have been the fact that initial ToM understanding was low and continued to be so throughout the study period.

The focus of longitudinal studies conducted to date has mostly been on early development of EF and ToM from toddlerhood up to the preschool years. Studies in older children are rare despite the importance of examining whether patterns found in early life remain throughout the course of the child’s development (McAlister and Peterson, 2013).

The few studies that have investigated the longitudinal relationship between EF and ToM in children beyond the preschool years show inconsistent results. A study by Farrant et al. (2012) in children aged 5 (at t1) found that early EF predicted later ToM, thus supporting Russell’s (1996) theory and replicating general findings of longitudinal studies in younger children. However, a 1-year longitudinal study with 5-year-old children showed that early EF did not predict later ToM (Razza and Blair, 2009). But EF at t2 was not assessed, so the reverse direction of early ToM predicting later EF could not be tested. A further study in 4-year-old children (at t1) even found the opposite, namely that early ToM predicted later EF even when controlling for age, language, siblings and initial EF (McAlister and Peterson, 2013), thus in line with Perner’s theory (Perner, 1998).

Taken together, longitudinal studies yielded mixed results on the exact nature of the causal relation between EF and ToM. If anything, there seems to be stronger evidence for Russel’s theory that EF is a prerequisite for ToM than for Perner’s theory that ToM supports EF (Russell, 1996, 1997; Perner, 1998).

The second type of evidence concerns dissociation studies. Perner’s and Russel’s theories make opposing predictions with respect to EF–ToM deficiencies. Perner’s account excludes the possibility that well-developed EF occurs paired with poor ToM: ToM is seen as a prerequisite for EF, and therefore ToM deficits should lead to impaired EF. But what is in line with his theory is the reverse pattern of well-developed ToM paired with EF deficits because intact ToM is necessary for EF, but not sufficient in its own right. On the other hand, Russel’s theory does not allow for deficits in EF to be paired with adequate ToM because his theory proposes that impaired EF leads to impaired ToM. His theory does permit the pattern of well-developed EF paired with deficits in ToM because the ToM impairment could have been caused by other factors, e.g., language, in particular inner speech (Pellicano, 2007).

Several dissociation studies indicate that EF is not required for the development of ToM, thus contradicting Russel’s theory. For example, a study conducted by Tager-Flusberg et al. (1997) and reanalyzed by Perner and Lang (2000) showed that children with Williams syndrome and Prader-Willi syndrome were impaired on EF tasks but mastered ToM tasks well. However, due to the small sample size (*N* = 6), results must be treated with caution. Studies on children diagnosed with ADHD showed a similar pattern, i.e., reasonably well-developed ToM skills paired with poor EF (Charman et al., 2001; Perner et al., 2002). However, it may well be that children in these studies mastered the ToM tasks in atypical ways, for instance by applying simple behavior rules (e.g., “doesn’t see, doesn’t know”; Garnham and Ruffman, 2001). If so, the relevance of EF for ToM would not be challenged by these results.

Other dissociation studies have found evidence contradicting Perner’s theory. Although autism typically involves low EF paired with low ToM (e.g., Hill, 2004), a study in 5½ year-old children with autism revealed a dissociation in exactly the opposite direction: a high level of EF with impaired ToM (Pellicano, 2007). Similarly, a cross-cultural study comparing U.S. and Chinese children aged about 4 years, controlled for age and verbal ability, found that although Chinese children had good executive control skills, their ToM understanding was poor (Sabbagh et al., 2006). Thus, evidence from dissociation studies revealed support for both Perner’s and Russel’s theories.

The third and final type of empirical support comes from training studies. For instance, a very recent study revealed the importance of EF for the improvement in the development of ToM. Preschool-aged children (aged 3 years 8 months) were given a battery of false-belief and EF tasks. Results showed that individual differences in initial EF predicted the degree to which children’s advances in ToM improved through ToM training, the relevant control variables being partialled out (Benson et al., 2013). However, a training study by Kloo and Perner (2003) did not support the view that early EF predicts later ToM to the same extent. They examined ToM via false-belief tasks, and attention shifting via the dimensional change card sort task (DCCS; Frye et al., 1995). Children (3- to 4-year-olds) were trained in these tasks about once a week over a period of weeks. Transfer of training took place in both directions: training on the DCCS task improved performance on the false-belief task, and false-belief training produced improvements on the DCCS task. This finding is in support of the idea of functional dependency between EF and ToM. However, it is interesting to note that training on the false-belief task did not lead to an improvement in post-training false-belief performance, which makes the interpretation of these findings problematical.

To sum up the three types of evidence, the general finding (particularly from longitudinal studies) in children younger than 4 years is that early EF predicts later ToM, and not vice versa. However, studies in older children have revealed inconsistent results. More research is needed to clarify the causal direction between EF and ToM in older children. In particular, possible differential relations between EF subcomponents and ToM are understudied to date. Therefore, the second aim of the present study was to examine whether each of the three EF subcomponents (attention shifting, WM updating, inhibition) at t1 predicted ToM at t2 (1 year later), or vice versa, in elementary school-aged children.

The current study investigated three subcomponents of EF (i.e., inhibition, attention shifting, WM updating) as well as ToM in a large sample of 6- to 12-year-old children. Moreover, we examined relations between EF and ToM controlled for age, gender and fluid intelligence. All tasks were administered at two measurement points which were about 1 year apart. Based on the evidence reviewed so far, we hypothesized first, the EF–ToM relationship to pertain in middle childhood. Thus, positive correlations should occur between EF and ToM performance in our sample. More specifically, we hypothesized that the three EF subcomponents and ToM at t1 are as strongly correlated as 1 year later at t2. Second, we expected to find relationships leading from EF subcomponents-t1 to ToM-t2, and not vice versa.

To our knowledge, this is the first study that addresses these issues in a representative sample of children in middle childhood.

## MATERIALS AND METHODS

### PARTICIPANTS

At t1 (in 2012), the sample consisted of *N* = 1,657 children (52% girls) aged between 6 and 11 years (*M* = 8.3 years, *SD* = 0.95). Time point 2 (t2; in 2013) took place 1 year later (*N* = 1,619) and children’s age ranged from 7 to 12 years (*M* = 9.1 years, *SD* = 0.92).

Participants were recruited from 33 primary schools from the federal state of Brandenburg, Germany. To establish a representative sample, schools were preselected so that participants coming from different rural and urban areas and socio-economic backgrounds were included. Before the study began, approval of all procedures was granted by the Research Ethics Committee at the University of Potsdam and the Ministry of Education, Youth, and Sports of the Federal State of Brandenburg. For each child, informed consent was obtained from the parent/primary caregiver. As a reward the children received a voucher for the cinema.

### MATERIAL

#### EF tasks

The EF subcomponent attention shifting was assessed using the Cognitive Attention shifting task (Röthlisberger et al., 2010; adapted from Zimmermann et al., 2002). Children were presented with a single-colored fish and a multi-colored fish appearing simultaneously on the left- and right-hand side of a computer screen. Participants were told to “feed” each kind of fish, alternating between the two, by pressing one of two keys on a QWERTZ keyboard (i.e., the X-key for the fish on the left, the M-key for the fish on the right). Across several trials, the side on which the two kinds of fish appeared changed randomly, requiring the children to remember their response of the previous trial in order to maintain the requirement of alternating feeding. The task consisted of a total of 46 trials (interstimulus intervals ranged from 300 to 700 ms) that were separated by a short break during which positive feedback was given. The dependent variable was the number of correct responses for the 22 switch trials. Switch trials were those answers that required children to change their response pattern (i.e., from pressing left/right to left/left or right/right).

The EF subcomponent WM updating was assessed using the Digit-Span Backward task (Petermann and Petermann, 2007). Participants were told a sequence of digits which they had to verbally repeat in the reverse order. The first sequence was two digits long. There were two sequences of equal length in each trial. Within each trial, at least one sequence had to be answered correctly in order to proceed to the next trial in which the sequence was lengthened by one digit. The dependent variable was the total number of sequences that had been remembered correctly.

The EF subcomponent inhibition was assessed by the Fruit Stroop task (Archibald and Kerns, 1999; adapted by Röthlisberger et al., 2010). The task consisted of four trials. For each trial, a page depicting 25 stimuli was presented to the child with the instruction to name the colors of the items as quickly as possible. Page 1 showed colored rectangles (blue, red, green, yellow). Page 2 depicted fruits or vegetables in their typical colors (banana – yellow, lettuce – green, strawberry – red, plum – blue). Page 3 showed the same fruits but these were colored gray. Page 4 showed the same fruits and vegetables, but all colored incorrectly. For pages 3 and 4, the children had to name the color that the fruit and vegetables should have had (i.e., banana – yellow, lettuce – green, etc.). The time (in seconds) required for naming the colors of all 25 items per page was measured. As dependent variable, an interference score was generated: time p.4 – [(time p.1 × time p.3)/(time p.1 + time p.3); Archibald and Kerns, 1999)]. Scoring high on this task is an indication of a lower ability to successfully inhibit the prepotent response of naming the items’ actual colors on page 4.

#### ToM task

**FIGURE 1:**
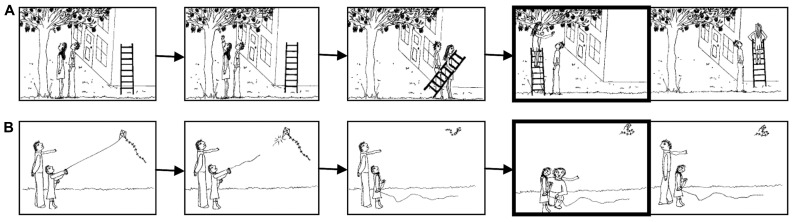
**Five pictures of one cartoon story for cognitive ToM (A) and affective ToM (B)**. The first three pictures appeared on a computer screen consecutively, the last two pictures appeared simultaneously. Children were instructed to select the correct ending by pressing a key. For illustration, the frame of the correct ending is marked black.

Theory of mind ability was assessed by a cartoon task developed by Völlm et al. (2006) for adults and adapted by Sebastian et al. (2012) for adolescents. The cartoon scenarios were presented on a computer screen. Each story consisted of five pictures with black-and-white line drawings depicting two individuals in order to control for social content (**Figure 1**). The first three story frames appeared consecutively, followed by two pictures shown simultaneously that displayed possible endings of the story. Children were instructed to choose the correct ending by pressing the X-key on a QWERTZ keyboard for the left-hand side, and the M-key for the right-hand side picture. In order to give a correct answer, children had to infer the mental state of one protagonist and the appropriate response by the other protagonist. Interstimulus intervals ranged from 1,000 to 3,000 ms. The order of the cartoons and the location of the correct ending were randomized across participants. Correct responses for each story were coded as 1, incorrect responses as 0.

The original task by Sebastian et al. (2012) consisted of 30 cartoons, 10 in each of three conditions: cognitive ToM, affective ToM, and a physical control condition. The physical control condition was used as baseline in order to determine neuronal activity in an functional magnetic resonance imaging (fMRI) setting. Because the current study did not use fMRI, and a baseline condition was not relevant, the physical condition was excluded. The remaining 20 cartoons (10 cognitive, 10 affective) were pretested for clarity, difficulty, and timings in children who were of similar age to those in our study and did not participate in the present study. Twelve cartoons (six cognitive ToM, six affective ToM) of mid-range difficulty were selected in order to limit the time demands of the task and to ensure sufficient variability in the data.

Each child was presented with six cognitive and six affective ToM stories. To choose the correct ending for the cognitive scenarios, children had to infer the appropriate behavior of one protagonist (e.g., helping) given the inferred intentions, desires, or beliefs of the other protagonist (e.g., attaining an action goal; **Figure 1A**). For the affective scenarios, participants needed to infer the appropriate response (e.g., consoling) of one protagonist regarding the emotional state (e.g., fear, sadness) of the other (**Figure 1B**). Performance on cognitive and affective trials was highly associated in our study (at t1: *r* = 0.83, *p* ≤ 0.001; at t2: *r* = 0.94, *p* ≤ 0.001). Thus, for reasons of simplicity and in line with the focus of the current paper, a model that did not differentiate between cognitive and affective aspects was chosen for further analyses. This one-factor model fitted the empirical data well at both time points, t1: χ^2^(9) = 17.11, *p* < 0.05, *CFI* = 0.99, *RMSEA* = 0.024, *WRMR* = 0.765, t2: χ^2^(9) = 14.85, *p* = 0.095, *CFI* = 0.988, *RMSEA* = 0.020, *WRMR* = 0.650. However, two items were excluded due to factor loadings falling under a general cutoff value (0.40) for the inclusion into one factor (Stevens, 2001).

In addition, the final version of the task was validated in a sample of 7.5- to 10-year-old children (*N* = 62, *M* age = 8.23, *SD* = 0.59, 35.5% girls) in order to ensure its association with standard ToM measures.

The standard ToM measures employed were two second-order false-belief tasks (Perner and Wimmer, 1985; Hollebrandse et al., 2012), a German version of the Extended Theory-of-Mind Scale (Henning et al., 2012) and German translations of the Strange Stories Test (Happé, 1994). Results showed that the total score of the standard ToM measures was positively correlated with the ToM cartoons total score (affective and cognitive cartoons combined) after controlling for language (*r* = 0.29, *p* = 0.025) or fluid intelligence (*r* = 0.32, *p* = 0.047).

#### Fluid intelligence

To measure fluid intelligence the Number-Symbol Test of the German version of the Wechsler Intelligence Scale for Children was used (Petermann and Petermann, 2007). The child was instructed to redraw symbols (e.g., a half moon) that were paired with either simple figures (e.g., a cross with a circle inside; Version A for 6- to 7-year-olds) or digits (1–9; Version B for 8- to 16-year-olds) as quickly as possible within 120 s. For both versions A and B, the dependent variable was the amount of correct symbols allocated within 120 s. For version A, extra points could be achieved if participants finished the task before the 120 s were over.

### PROCEDURE

At both t1 and t2, children were assessed for two 50-min sessions spaced 1 week apart. Assessments were part of a larger battery of tasks that were conducted separately with each child by a specifically trained PhD student or research assistant in a quiet room either in a school setting or at home. The order of the larger battery of tasks was counterbalanced across participants (blocks of ABCD/BADC) but the order did not show any effect when subsequently analyzed.

### DATA ANALYSIS

All analyses were run using Mplus 7.11 (Muthén and Muthén, 1998–2012). The rate of missing data was low on all variables at t1 (≤1.8) and at t2 (≤7.2). Assuming data to be missing at random, all missing values were accounted for by full information maximum likelihood (FIML) estimation.

Research questions were answered by means of structural equation modeling (SEM). As mentioned above, model fit was considered acceptable only if absolute fit indices fulfilled the following criteria: *CFI* ≥ 0.95, *RMSEA* ≤ 0.08, *WRMRV* ≤ 1.0 (Yu, 2002; Geiser, 2010).

The cartoon stories were entered in the analyses as categorical indicators (1 for choosing the correct ending, 0 for an incorrect choice). As the χ^2^ depends on sample size and is overly sensitive to deviations from perfect fit in large samples (Schermelleh-Engel et al., 2003).

In order to answer the first research question (the extension of the EF–ToM relationship to middle childhood) the concurrent correlations between each EF subcomponent and ToM at t1 and t2 were examined (EF-t1 – ToM-t1 and EF-t2 – ToM-t2). In addition, the correlation coefficients at t1 and t2 were statistically compared in order to determine whether the EF–ToM association was equally strong at each time point. This procedure was followed for each of the three EF subcomponents in order to detect possible differential relations.

To answer the second research question (the longitudinal relations between EF and ToM over a 1-year period), three cross-lagged models were fit to the data, describing the assumed interrelations between each EF subcomponent and ToM over time. By controlling for initial levels of the target variable, cross-lagged models ensure that the association of one variable as developmental precursor of another variable is examined, rather than concurrent associations (McAlister and Peterson, 2013). Subsequently, regression coefficients for the cross-lagged paths were compared in order to evaluate for which direction the association was stronger, i.e., ToM-t1 to EF-t2 or EF-t1 to ToM-t2.

In all analyses, ToM was entered as latent and the three EF subcomponents (attention shifting, WM updating and inhibition) as manifest variables. Moreover, fluid intelligence, age and gender which are known to be related to EF and ToM were included as manifest control variables.

## RESULTS

### DESCRIPTIVES

Descriptive statistics of the assessed variables are shown in **Table 1**. At both measurement time points, medium scores were achieved on WM updating, inhibition and fluid intelligence, medium to high scores on attention shifting and high scores on ToM. Intercorrelations between the three EF subcomponents ranged from 0.27 to 0.35 (all *p*s ≤ 0.001) at t1 and from 0.28 to 0.33 at t2 (all *p*s ≤ 0.001). On average, participants improved in the EF and ToM tasks from t1 to t2, indicating a significant developmental change in those abilities within a year.

**Table 1:** Descriptive statistics of assessed variables (EF subcomponents attention shifting, WM updating, inhibition, and ToM cartoon task) for the two measurement time points (t1 and t2) and mean comparison results.

	t1			t2			*t*
	*M*	*SD*	Min.–max.	*M*	*SD*	Min.–max.	
Attention shifting	15.6	4.7	0–22	18.1	3.9	0–22	-24.2^***^
WM updating	6.1	1.6	0–16	6.6	1.5	0–16	-11.1^***^
Inhibition^a^	24.9	8.8	0–89^b^	20.6	6.9	0–66^b^	^23.2***^
ToM^c^	8.5	1.7	0–10	9.2	1.3	1–10	-14.5^***^
Fluid intelligence^d^	51.4	9.2	27–80^b^	/	/	/	/

a ToM, theory of mind; ^a^interference measure (negatively polarized); ^b^min and/or max values are theoretically infinite, thus table values are sample-specific; ^c^average number of correct trials; ^d^only the t1 measurement of fluid intelligence was included in the analysis; ***p ≤ 0.001.

### RESEARCH QUESTION 1: CONCURRENT ASSOCIATIONS BETWEEN EF SUBCOMPONENTS AND ToM

The first research question concerned the concurrent correlations at both time points controlled for age, gender, and fluid intelligence, that is, the association between EF subcomponents and ToM at t1 and t2 (see **Table 2**). At both time points, correlations were small but significant (ranging from 0.10 to 0.20; Cohen, 1988) indicating that EF subcomponents and ToM were associated in 6–11 year-olds and in 7–12 year-olds. Testing the strength of the concurrent path coefficients against each other showed that for each EF subcomponent the difference of t1 and t2 concurrent correlations with ToM was not significant (attention shifting: Δχ^2^ (1) = 0.041, *p* = 0.84; WM updating: Δχ^2^ (1) = 3.285, *p* = 0.07; inhibition: Δχ^2^ (1) = 0.037, *p* = 0.85).

**Table 2:** Concurrent correlations between EF subcomponents and ToM at t1 and t2, respectively, controlled for age, gender, and fluid intelligence.

	ToM-t1		ToM-t2
Attention shifting-t1	0.19^**^	Attention shifting-t2	0.20^**^
WM updating-t1	0.13^**^	WM updating-t2	0.20^**^
Inhibition-t1^a^	-0.08^*^	Inhibition-t2	-0.10^**^

a Interference measure (negatively polarized); **p ≤ 0.01, *p ≤ 0.05, two-tailed.

### RESEARCH QUESTION 2: RECIPROCAL INFLUENCES BETWEEN EF SUBCOMPONENTS AND ToM ACROSS 1 YEAR

**FIGURE 2:**
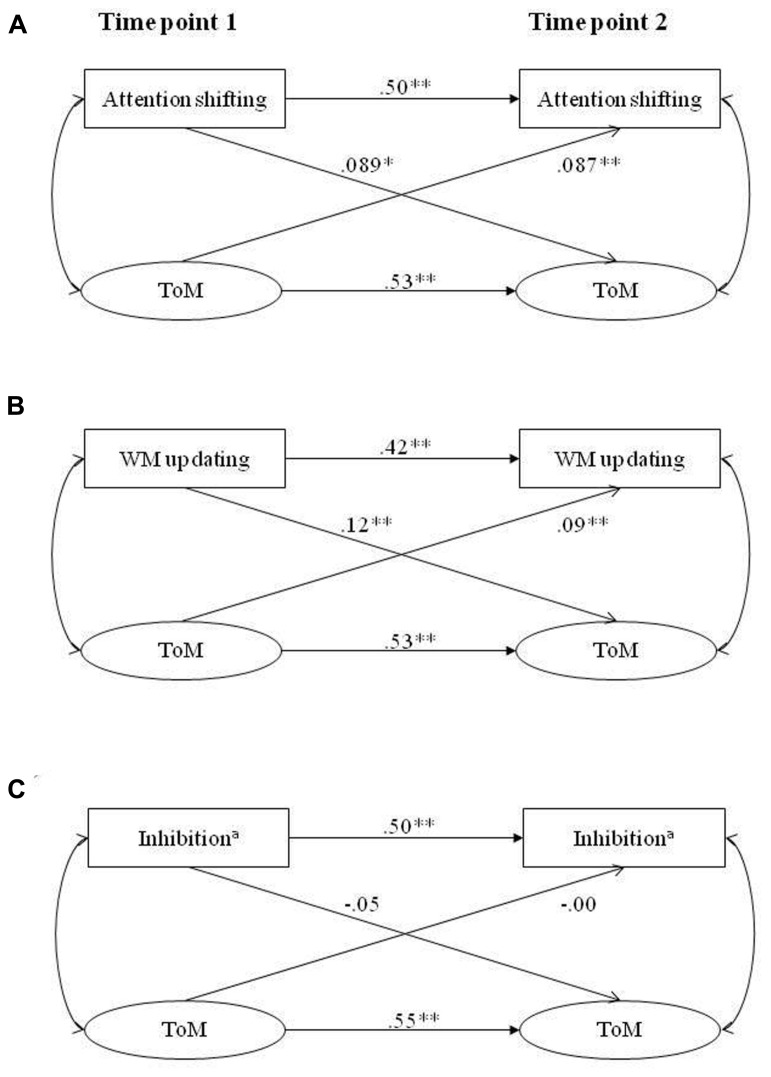
**Cross-lagged models for the relations between ToM and the EF subcomponents attention shifting (A), WM updating (B), and inhibition (C), at the two measurement points, controlling for age, gender, fluid intelligence, as well as for the earlier levels of the target variables**. ^a^Interference measure (negatively polarized); **p ≤ 0.01, *p ≤ 0.05, two-tailed.

**Figure 2** shows the cross-lagged models for the interrelation between each EF subcomponent and ToM over time controlled for initial levels of the outcome variable at t1 as well as for the covariates age, gender, and fluid intelligence.

The model for attention shifting (**Figure 2A**) fitted the data well, χ^2^ (259) = 321.13, *p* = 0.005, *CFI* = 0.98, *RMSEA* = 0.012, *WRMR* = 0.90. Both cross-lagged path coefficients (attention shifting-t1 to ToM-t2 and ToM-t1 to attention shifting-t2) reached significance. Despite the high autocorrelations of attention shifting and ToM from t1 to t2 (**Figure 2A**), and after controlling for age, gender, and fluid intelligence, the cross-lagged paths still revealed a small but significant association of EF on ToM, and vice versa, across the 1-year period. Testing the strength of the cross-lagged path coefficients against each other showed a significant difference, Δχ^2^ (1) = 7.999, *p* < 0.01, with a stronger association leading from attention shifting-t1 to ToM-t2 than in the opposite direction.

The model for WM updating (**Figure 2B**) also fitted the data well, χ^2^ (259) = 328.75, *p* = 0.002, *CFI* = 0.98, *RMSEA* = 0.013, *WRMR* = 0.91. Again, both cross-lagged path coefficients (WM-t1 to ToM-t2 and ToM-t1 to WM-t2) revealed a small, but significant impact over and above the high autocorrelations over time and possible effects of the control variables. The difference between the cross-lagged path coefficients was marginally significant, Δχ^2^ (1) = 3.42, *p* = 0.064, with a stronger association leading from WM-t1 to ToM-t2 than in the opposite direction.

The model for inhibition (**Figure 2C**) also fitted the data well, χ^2^ (259) = 316.28, *p* = 0.009, *CFI* = 0.98, *RMSEA* = 0.012, *WRMR* = 0.90. However, for this model neither of the cross-lagged path coefficients reached significance. Thus, for the EF subcomponent inhibition no significant cross-lagged relationships with ToM over time were found.

## DISCUSSION

The present study pursued two objectives. First, we examined whether the relationship between EF and ToM pertains in middle childhood (6–12 years). Results showed small but significant concurrent correlations in the expected directions between all studied EF subcomponents (attention shifting, WM updating and inhibition) and ToM. In line with previous research (Perner et al., 2002; Yang et al., 2009; Calderon et al., 2010), better abilities in executive control of thought or action were related to better understanding of others’ mental states at t1 (6–11 years) and t2 (7–12 years). Second, we explored whether each EF subcomponent at t1 predicted ToM at t2, or vice versa, over a 1-year period. Here, we used a cross-lagged model, again controlling for age, gender and fluid intelligence, as well as for the earlier levels of the target variables. Results showed small, but significant bidirectional longitudinal relationships with ToM for two EF subcomponents, WM updating and attention shifting. Examining the strength of the associations showed that for both EF subcomponents, the relationship between early EF and later ToM was stronger than the relationship between early ToM and later EF. Bearing in mind that effect sizes were small this corresponds with research on longitudinal studies between EF and ToM in preschool-age children (e.g., Hughes, 1998b) and illustrates the pertaining relevance of the ability to switch between different task demands and the ability to temporarily hold information in mind while processing it for developing ToM abilities. For the subcomponent inhibition, however, no reciprocal relationships were found over time. Thus, discriminating between EF subcomponents seems to be important for the study of EF–ToM development, because on the one hand, the single EF subcomponents may follow different developmental courses and on the other hand, understanding the mental states of others at various levels (e.g., first- or second-order perspective) may put different demands on EF subcomponents.

### THE RELATIONSHIP BETWEEN THE THREE EF SUBCOMPONENTS AND ToM IN MIDDLE CHILDHOOD

The relationship between EF subcomponents and ToM is well documented in the preschool years (e.g., Frye et al., 1995; Hughes, 1998a; Carlson and Moses, 2001). However, still in need of clarification is the question how exactly this relationship extends to middle childhood for each of the EF subcomponents. To date, there are few studies in children beyond the preschool age, and, as we will discuss below, those conducted are inconclusive. Results of the present study indicate that the relationship between all three EF subcomponents (attention shifting, WM updating, inhibition) and ToM pertain in middle childhood with small, but significant correlations (age, gender, and fluid intelligence partialled out).

In a sample of similar-aged children (8.5-year-olds) Charman et al. (2001) also found a correlation between EF (GoNoGo error score) and ToM (Happé stories correct mental score) for their typically developing control group (*r* = -0.43, *p* < 0.01). However, as soon as age and intelligence were partialled out, their results fell below significance (*r* = -0.38, *p* = 0.10). This may be owing to the low statistical power due to the relatively small size of their control group (*N* = 22). The diverging results to the present study may also come from the use of different measures. Charman et al. (2001) assessed ToM by means of Happé’s Strange Stories (Happé, 1994), a measure of higher-order ToM ability. The Strange Stories task consists of naturalistic, short vignettes which are read to the child by the experimenter. The task makes strong demands on verbal ability. In contrast, in the present study, a ToM cartoon task was used. It is a non-verbal task that was originally developed for fMRI studies (Völlm et al., 2006). Each cartoon story is presented on a computer screen, and the correct answer is chosen by pressing a key. Because of the lower verbal task demands of the ToM cartoon test, children in the present study were probably not impaired in expressing their ToM ability.

Turning to EF, Charman et al. (2001) examined different subcomponents (planning and behavioral inhibition) compared to our study. Generally, the fact that different studies involve various aspects of EF makes comparisons between studies difficult. This inconsistency might be due to the fact that EF is an ill-defined construct that has been described as an umbrella term for a large array of different processes involved in self-control (Kerr and Zelazo, 2004). The EF definition applied in the current study follows Miyake et al.’s (2000) division into three overlapping but distinct subcomponents – attention shifting, WM updating, and inhibition. This approach has proved promising and has been adopted in studies on the relations between ToM and EF in preschool children.

Another problem when comparing different studies on EF–ToM relations is that the available tasks do not test only one EF subcomponent, but engage overlapping EF aspects to varying degrees. The planning tasks employed by Charman et al. (2001) displays this *task impurity* to a rather high degree because more than one EF subcomponent was needed to meet the relatively complex task requirements (Miyake et al., 2000). Because the present study used simpler tasks, which mainly required one EF subcomponent, children could probably express their EF capacities in an optimal way. This may be another reason why we, unlike Charman et al. (2001) still found small but significant EF–ToM relations in 6- to 12-year-olds after controlling for age, gender, and intelligence.

Our results are supported by a study in slightly younger children (4^1^/_2_–6^1^/_2_ year-olds) where strong relationships between several EF and ToM tasks were found even when controlling for age and IQ and despite the relatively small sample size (*N* = 22) of their control group (Perner et al., 2002). These similar findings can possibly be attributed to the fact that similar measures were implemented. Perner et al. (2002) used a second-order false-belief task (based on the material by Perner and Wimmer, 1985). We argue that at least 6 out of 10 of our ToM cartoon stories require second-order reasoning. Because the cartoons were originally used in a study with a different focus - differentiating ToM into cognitive and affective aspects - Sebastian et al. (2012) did not address the issue of first- or second-order ToM reasoning. What they did maintain was that the affective condition requires cognitive ToM. Yet, in the six affective ToM cartoon tasks, in order to give a correct response, the participant has to understand the first protagonist’s belief about the second protagonist’s mental state (e.g., the adult *believes/thinks* the child is sad because the child does not *want* the kite to fly away; i.e., second-order ToM). In the four cognitive ToM cartoon tasks, however, in order to choose the correct ending, the participant has to infer the desire of only one of the protagonists (e.g., the girl places the ladder against the tree because she *wants* an apple; i.e., first-order ToM). The second protagonist merely accompanies the first protagonist.

We also tested possible differences in the strength of the concurrent relationship between EF subcomponents and ToM at both time points. We had no reason to hypothesize that there would be any change in the EF–ToM relations in the space of 1 year in 6 to 12-year-olds. The present results showed precisely this, suggesting that all three EF subcomponents and ToM remain related, with not much change in the strength of the relations across a 1-year-period, in a representative sample in middle childhood.

### THE RECIPROCAL RELATIONSHIP BETWEEN EF SUBCOMPONENTS AND ToM

The second aim of the present study explored the longitudinal bidirectional relationship between the three EF subcomponents and ToM over a 1-year period. In a cross-lagged model, we again controlled for age, gender, and fluid intelligence, and also partialled out earlier EF or ToM performance. Our findings revealed small, but significant reciprocal relations with ToM for two EF subcomponents, attention shifting and WM updating, but no relations for inhibition. The lacking longitudinal relations between inhibition and ToM may indicate that this EF subcomponent is of less importance for ToM in middle childhood as compared to the preschool age, particularly over time. The different results for the three EF subcomponents underlines the necessity of examining EF development divided into its specific subcomponents. Interestingly, for both attention shifting and WM updating, the relationship of early EF predicting later ToM was stronger than the relationship of early ToM predicting later EF (the difference for WM updating was marginally significant). Although our effect sizes were small, this finding supports Russell’s (1996) theory that EF precedes ToM development and is in line with results of previous longitudinal studies on EF–ToM relationship in preschool-age children.

One of the earliest such studies found that in 3- to 4-year-old children (mean age: 3 years and 11 months) early EF performance predicted later ToM (13 months later) even when controlling for age, verbal ability, and initial ToM (Hughes, 1998b). The reverse direction was not found, which has been interpreted as evidence for Russel’s theory. However, only one of the EF measures, a detour-reaching box (Hughes and Russell, 1993), a measure of inhibitory control, showed an independent predictive relationship with later ToM in Hughes’s study. The other EF subcomponents (WM, planning, attention shifting) did not. Interestingly, in the present study with older children, the only two EF subcomponents that showed small but significant relationships with ToM over time were attention shifting and WM updating, but not inhibition. A possible explanation for this finding may be that preschool-age children are still in the course of developing their inhibitory skills and thus rely on these more heavily. This is reflected in medium to high longitudinal correlations between inhibition and ToM. In addition, it may also be that first-order false-belief tasks (as used by Hughes) make more demands on inhibitory skills compared to second-order ToM items (as mainly used in the present study). In first-order false-belief tasks, the child’s own knowledge about reality has to be inhibited in order to give a correct answer. To solve second-order ToM tasks, inhibition may play less of an important role, because the focus lies more on being able to switch flexibly between the different mindsets of the first and second protagonist (Miller, 2009). Also, WM updating is needed to keep track of the different perspectives and bringing all the relevant information together. The exact nature of the relationship between different EF subcomponents and ToM across childhood requires further research.

Just like Hughes’s (1998b) study, Carlson et al.’s (2004) longitudinal study in a younger group of children (2-year-olds) showed similar asymmetrical relationships: early EF predicted later ToM (15 months later) even when controlling for age, gender, and verbal ability. However, as mentioned in the introduction, Carlson et al.’s (2004) results must be interpreted with caution as ToM was assessed with different measures at the two time points and did not correlate over time.

Another longitudinal study by Hughes and Ensor (2007) examined the predictive relationship between EF and ToM at three time points in 2-, 3-, and 4-year-olds. Results showed only limited support for Perner (1998), Perner and Lang’s (1999) theory – that early ToM predicts later EF – but stronger support for Russell’s (1996) theory – that early EF predicts later ToM. One advantage of their study was that they included participants from a variety of social backgrounds. This issue has been neglected in previous research despite the fact that socioeconomic status (SES) is known to predict cognitive abilities (Bradley and Corwyn, 2002) and may have an impact on the EF–ToM relationship (Hughes and Ensor, 2007). Although only little is known about the impact of SES on EF or ToM development in middle childhood, the present study established a representative sample by preselecting schools from different socio-economic backgrounds in both rural and urban communities. Therefore, the present results are probably not affected by possible SES effects.

Turning now to our findings of reciprocity, although the present study showed stronger relationships leading from early attention shifting and WM updating to later ToM, one cannot dismiss the fact that the relationship was bidirectional. That is to say, there were indeed small, but significant relations in the opposite direction. We did not expect this finding because previous longitudinal studies almost consistently showed a unidirectional association in which early EF predicted later ToM and not vice versa (Hughes, 1998b; Carlson et al., 2004; Flynn et al., 2004; Jahromi and Stifter, 2008; Müller et al., 2012). However, in line with our findings, Hughes and Ensor (2007) also found partial support for early ToM – later EF, but stronger evidence for early EF – later ToM. Likewise, McAlister and Peterson (2013) found that ToM at 3 years 3 months to 5 years 6 months predicted EF at about 1 year later, but they did not find a significant relation in the opposite direction. Moreover, Kloo and Perner’s (2003) training study in 3- to 4-year-olds suggested a bidirectional relationship between EF and ToM because transfer of training took place in both directions. They took their results to support the idea of a functional dependence between the two constructs in that “understanding the mind presupposes a certain degree of executive control, and EF presupposes a certain level of insight into the mind” (Kloo and Perner, 2003, p.1836). It has been suggested elsewhere that the EF–ToM relationship can be interpreted in reciprocal terms with one construct complementing the other (Putko, 2009). However, as noted by Kloo and Perner (2003), their study cannot clarify the causes and processes involved that are responsible for this relationship. Both constructs may be related in an indirect way, that is, individuals with well-developed EF may be better equipped to function well in social groups and this then encourages improvements in ToM (Hughes, 2001). Further studies are needed to shed more light on the relationship between EF and ToM in reciprocal terms and on the exact interplay of the two constructs, especially for the age range beyond the preschool years.

In sum, although a few studies have shown that early ToM predicts later EF, the majority of longitudinal studies conducted so far reveal more evidence for the view that early EF predicts later ToM. However, the existing longitudinal research has almost exclusively focused on the preschool or late toddlerhood years. To our knowledge, the current study is the first to find longitudinal support suggesting that early EF subcomponents predict later ToM in a representative sample in middle childhood. This is an interesting finding as it suggests that although sufficient cognitive capacities have developed, a higher level of EF (WM updating and attention shifting) continues to be associated with a higher level of ToM. Thus, especially the ability to switch between different task demands and the ability to temporarily hold information in mind while processing it seem to be important for understanding mental states in middle childhood. The importance of these two EF subcomponents is reflected in second-order false-belief tasks, the most commonly used ToM task in older children. In order to make a correct inference on this task, the different perspectives of the mindsets of the two protagonists need to be taken into account (attention shifting) and, in addition, all the pieces of information need to be actively held in mind and updated (WM updating). Thus it appears that while children progress through childhood and their social contexts become increasingly complex (e.g., school entry in which friendships and relationships to peers and teachers are formed), attention shifting and WM updating are needed for the children to make sense of and function within their social surroundings.

### LIMITATIONS AND OUTLOOK

A problem which has been discussed in the literature is the possible difficulty that children may have to express their existing ToM abilities due to the EF requirements inherent in the ToM task (Carlson and Moses, 2001; Moses, 2001). Referring to the instruments in our study, it is possible that our ToM cartoon requires EF demands which may explain the concurrent as well as the longitudinal correlations between the two constructs. However, several arguments speak against this. Many children completed our ToM cartoon task successfully, which implies that the EF demands were not overly high. In addition, it may be that our ToM cartoon task measures WM capacity, producing a task impurity problem (Miyake et al., 2000). For each cartoon, three pictures were shown consecutively, which had to be remembered by the child in order to choose the correct ending. But because the pictures were presented in quick succession, they resembled a cartoon film strip with an easy script, not making overly high demands on WM capacity. However, even if the latter were to be the case, children were not required to mentally process or re-arrange the pictures in any way. Therefore, these potential task demands cannot explain the relations with updating of WM which was one of our EF subcomponent.

Another argument on the same lines is that the relation between attention shifting and ToM may result from the fact that both tasks require shifting between pressing the left and right key. However, the ToM cartoons did not call for shifting between different answer sets or for abandoning an acquired rule of alternating between pressing the left and right key (as the attention shifting task did). In the 10 ToM cartoons, children just had to decide which of the two presented pictures displayed the correct ending, and they had ample time to press the appropriate key. The high rate of correct responses for the ToM cartoons indicates that this task was rather easy for the children. Therefore, we take the relations between attention shifting and ToM as evidence that our ToM tasks required attention shifting with respect to inferring the mental states of the two displayed protagonists (rather than with respect to the task design).

Second, further studies on longitudinal relations between EF subcomponents and ToM in middle childhood should include more than two time points. This would not only show how the EF–ToM relationship develops over a longer period, but also allow an investigation of moderating or mediating factors.

Furthermore, although our study yielded innovative findings, it would be interesting to use more than one ToM and more than one task for each EF subcomponent. Using more measures would no doubt increase reliability, and it would shed further light on relations between EF subcomponents and different aspects of ToM that emerge in middle childhood (e.g., second-order false-belief, irony, contrary emotion; Happé, 1994).

In conclusion, the current study suggests that the relationship between three EF subcomponents (attention shifting, WM updating, inhibition) and ToM pertains in a representative sample in middle childhood. Partial evidence was provided for the assumption that early ToM predicts later EF (Carruthers, 1996; Perner, 1998; Perner and Lang, 1999, 2000) but there was stronger support for the proposal that early EF (attention shifting, WM updating) predicts later ToM (Russell, 1996). In addition, our results suggest a reciprocity in the EF–ToM relationship over a 1-year-period. Future studies are needed to shed more light on the precise interplay of the two constructs, especially with respect to subcomponents of both EF and ToM, in the course of childhood development.

## Conflict of Interest Statement

The authors declare that the research was conducted in the absence of any commercial or financial relationships that could be construed as a potential conflict of interest.
